# Standing Still: A Case of Stiff Person Syndrome and Common Variable Immunodeficiency

**DOI:** 10.7759/cureus.96760

**Published:** 2025-11-13

**Authors:** Vicken Khazar, Samuel Escobar, James Kim, Steve McClain, Anthony M Szema

**Affiliations:** 1 Medicine, Donald and Barbara Zucker School of Medicine at Hofstra/Northwell, Hempstead, USA; 2 Allergy and Immunology, Pulmonary Medicine, Three Village Allergy and Asthma, PLLC, Centereach, USA; 3 Medicine, Warren Alpert Medical School of Brown University, Providence, USA; 4 Pathology, McClain Laboratories LLC, Smithtown, USA; 5 Allergy and Immunology, Pulmonary Medicine, Donald and Barbara Zucker School of Medicine at Hofstra/Northwell, Hempstead, USA

**Keywords:** anti-gad antibodies, common variable immunodeficiency (cvid), fungal pneumonia, recombinant human hyaluronidase ph20, rituximab therapy, stiff-person syndrome

## Abstract

Stiff-person syndrome (SPS) is a rare autoimmune neurologic disorder characterized by progressive rigidity and spasms, while common variable immunodeficiency (CVID) features hypogammaglobulinemia and recurrent infections. Their coexistence complicates management by requiring autoimmune suppression without further compromising host defense. A 54-year-old man with CVID, diagnosed based on hypogammaglobulinemia (IgG 386 mg/dL) and recurrent sinopulmonary infections, subsequently developed SPS with progressive truncal and limb stiffness, causing gait impairment and dyspnea. He experienced a severe aspergillus pneumonia, and examination showed finger clubbing and resting hypoxemia. Pulmonary function testing demonstrated moderate airflow obstruction with distal airway involvement. Chest radiography later revealed an elevated left hemidiaphragm, consistent with respiratory muscle dysfunction in SPS. Combined therapy with intravenous immunoglobulin (IVIG) and rituximab was initiated, yielding meaningful improvement in stiffness and a reduction in respiratory infections. After a motor vehicle accident with spinal fusion, the patient reported worsening exertional dyspnea and variable IgG troughs; treatment was transitioned to subcutaneous immunoglobulin (SCIG) 10% with rHuPH20 to maintain steadier IgG levels. This case emphasizes the importance of immunologic evaluation in refractory SPS and demonstrates that combined IVIG and rituximab can provide functional benefit while addressing CVID. Transition to SCIG may stabilize IgG exposure and sustain clinical gains. A timeline of diagnoses and treatments highlights the interplay between autoimmunity and hypogammaglobulinemia and supports a tailored, multidisciplinary strategy.

## Introduction

Stiff-person syndrome (SPS) is a rare, progressive, immune-mediated disorder of the central nervous system characterized by axial and proximal limb rigidity with painful spasms and exaggerated startle. Autoimmune B-cell activity against GABAergic pathways, often anti-glutamic acid decarboxylase (anti-GAD) antibodies, disrupts inhibitory signaling and produces cortical hyperexcitability [1-4]. Classic SPS commonly begins insidiously in the thoracolumbar region, progressing to gait disturbance, falls, orthopedic deformities, and neuropsychiatric sequelae [5,6]. Immunotherapies such as intravenous immunoglobulin (IVIG) and, in selected cases, rituximab, can reduce exacerbations, although disease-modifying targets remain uncertain [6-8].

Common variable immunodeficiency (CVID) is a primary humoral immune disorder defined by low IgG with low IgA and/or IgM and impaired antibody responses, leading to recurrent sinopulmonary infections, autoimmunity, chronic lung disease, and increased malignancy risk [9,10]. Immunoglobulin replacement is the cornerstone of therapy, yet infusion reactions and variability in IgG troughs complicate long-term management [10].

Co-occurrence of SPS and CVID is clinically significant for two reasons. First, B-cell-directed and immunoglobulin-based therapies used for SPS can simultaneously address or aggravate humoral immunity; balancing autoimmune control against infection risk is challenging. Second, SPS may contribute to respiratory compromise (e.g., diaphragmatic dysfunction) in a patient already vulnerable to infections due to CVID, amplifying pulmonary morbidity.

We present a patient with coexisting SPS and CVID in whom combined IVIG plus rituximab provided dual benefit-improving SPS symptoms while reducing infections. We also describe a strategic transition to subcutaneous IgG with rHuPH20 to stabilize IgG exposure when trough variability and respiratory infections increased. We propose a practical management framework that integrates immunologic monitoring, targeted B-cell therapy, and route optimization of immunoglobulin replacement in SPS with concomitant antibody deficiency.

This report was previously presented as a poster at the 2023 American Thoracic Society International Conference (May 21, 2023).

## Case presentation

A 54-year-old caucasian man was diagnosed with CVID in 2021 based on hypogammaglobulinemia (IgG 386 mg/dL; reference, 700-1600 mg/dL) and recurrent bacterial sinusitis and pneumonias. In March of 2022, he developed progressive truncal and proximal limb stiffness, spasms, gait impairment, and startle, consistent with SPS. Relevant history included chronic obstructive pulmonary disease (COPD) (20 pack-year smoking history), occupational exposure to epoxy/fumes, deep vein thrombosis, myocardial infarction, and obstructive sleep apnea. He reported dyspnea at rest and when supine, poor sleep due to spasms, and difficulty walking one block or lifting his newborn child. Examination showed coarse breath sounds, digital clubbing, and oxygen saturation of 90% on a 2 L/minute nasal cannula. Spirometry demonstrated a moderate obstructive ventilatory defect; impulse oscillometry indicated distal airway narrowing and airway hyperresponsiveness (Table 1).

**Table 1:** Spirometry indicates obstructive ventilatory defect, and impulse oscillometry testing shows airways hyper-responsiveness and distal airways narrowing. FVC: forced vital capacity; FEV1: forced expiratory volume in one second; R5Hz: resistance to airflow measured at a frequency of 5 Hertz; R20Hz: resistance to airflow measured at a frequency of 20 Hertz; X5Hz: reactance of peripheral airways at 5 Hertz; fRES: resonant frequency; R5-20%: percent difference between resistance to airflow measured at a frequency of 20 Hertz and resistance to airflow measured at a frequency of 5 Hertz

Pulmonary Function Test	Predicted value	Actual value	Percent of predicted value (%)
Spirometry			
VC Max (L)	3.89	2.81	72
FVC (L)	3.89	2.81	72
FEV1 (L)	3.02	1.53	51
FEV1/FVC (%)	77	54	70
Impulse Oscillometry			
R5Hz (cmH₂O/(L/s))	2.96	4.28	144
R20Hz (cmH₂O/(L/s))	2.55	3.10	122
X5Hz (cmH₂O/(L/s))	-0.06	-1.56	2576
fRES 1/s	-	22	-
R5-20%	-	38.03	-

**Figure 1:**
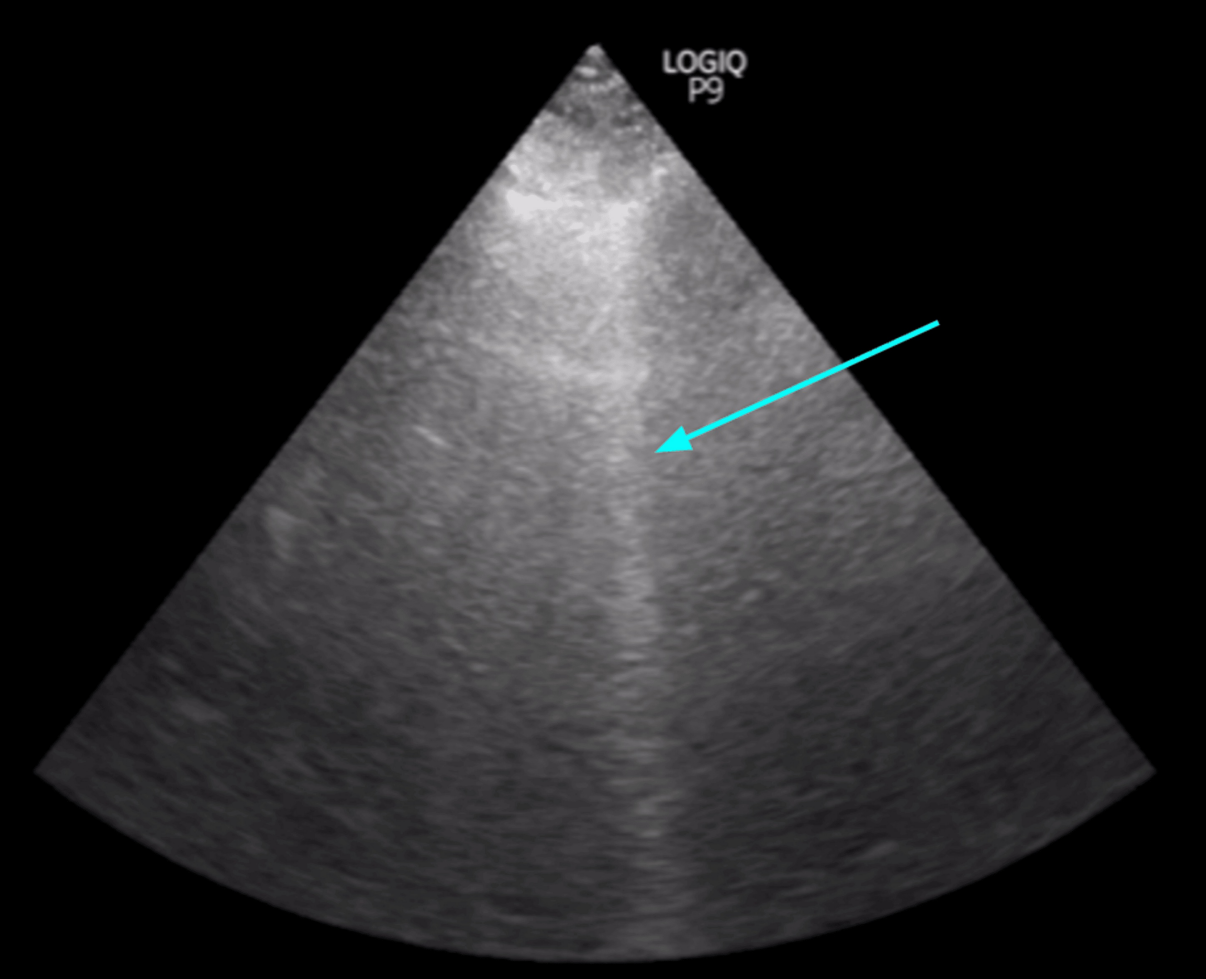
Chest ultrasonography showing prominent B-line (teal arrow) without pleural effusion at the right lung base.

In September of 2022, he was hospitalized for 11 days with fever, hemoptysis, and dyspnea; workup confirmed *Aspergillus* pneumonia (positive beta-D-glucan), and he improved on intravenous voriconazole. He developed hyperammonemia (>80 μM) during admission, treated with lactulose. Point-of-care lung ultrasound showed right basal B-lines without pleural effusion (Figure 1).

**Figure 2:**
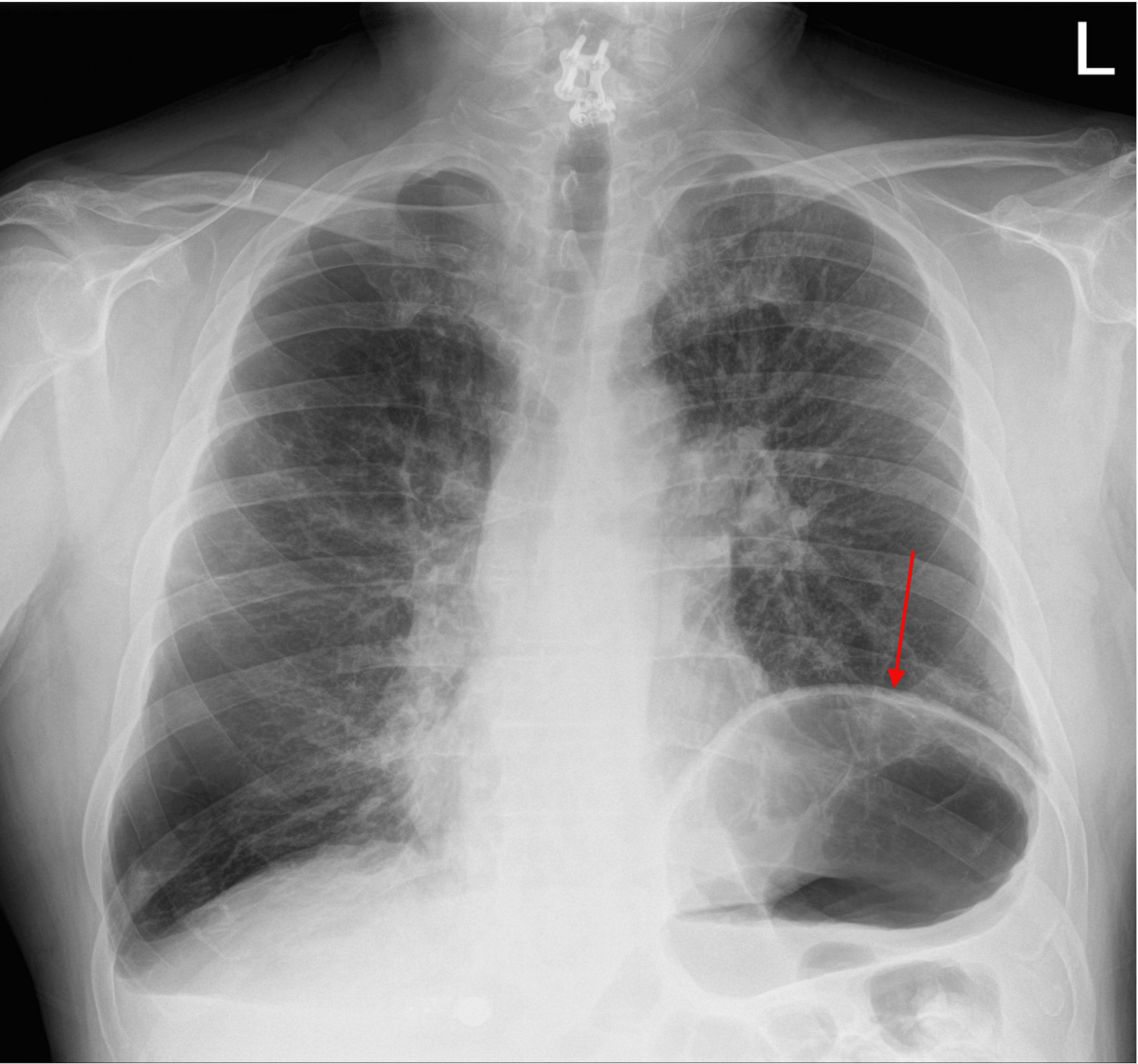
Chest radiograph showing elevated left hemidiaphragm (red arrow), consistent with presentation of dyspnea and history of SPS. SPS: stiff-person syndrome

On the three-day post-hospitalization outpatient follow-up, he had increased fatigue and exertional dyspnea. ECG showed left axis deviation, right bundle branch block, and premature ventricular contractions, without acute ischemia. Chest radiography revealed an elevated left hemidiaphragm, supporting a component of respiratory muscle dysfunction in SPS (Figure 2).

**Figure 3:**
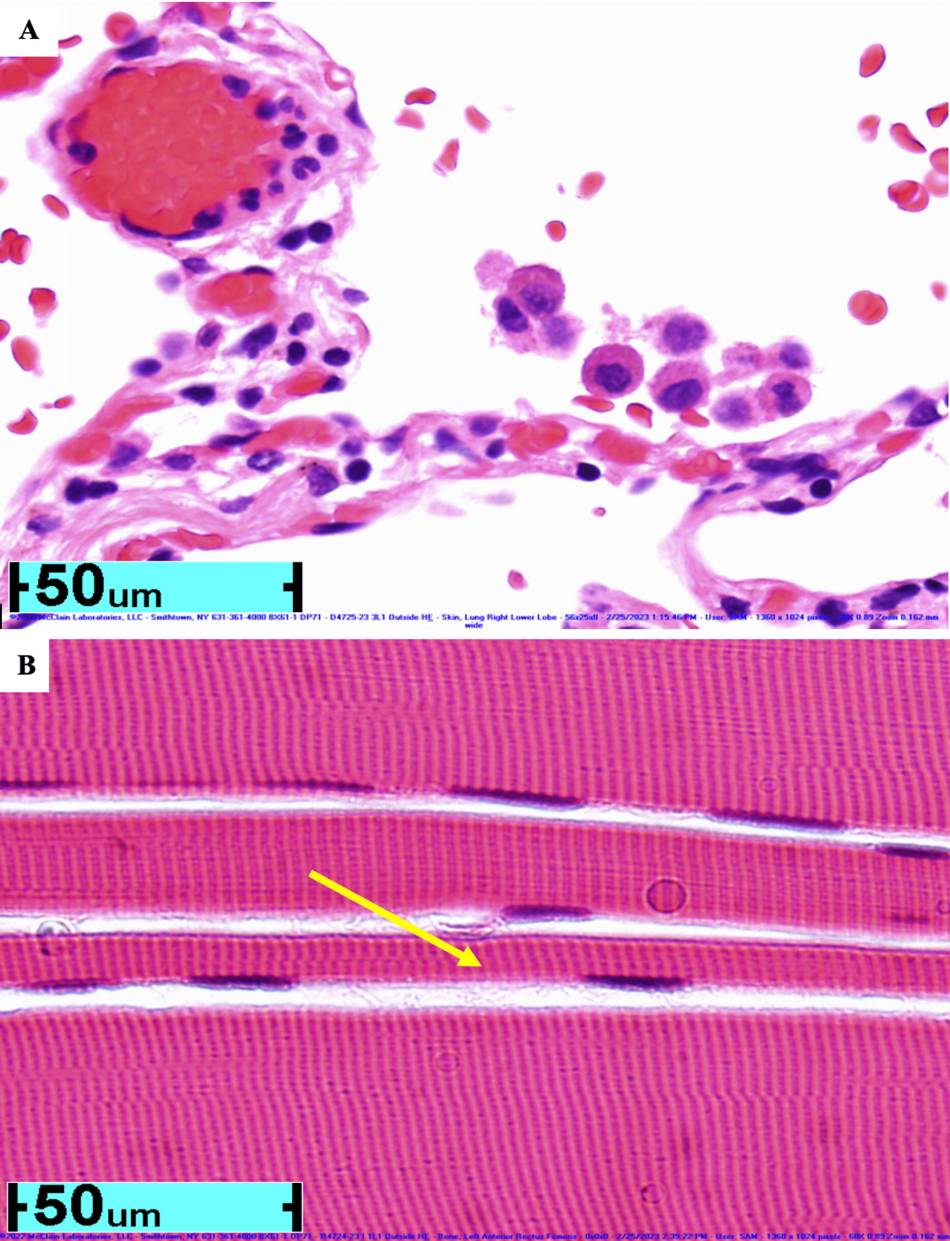
(A) Histopathology of right lower lung wedge biopsy showing diffuse degeneration of an alveolar wall consistent with COPD. (B) Histopathology of leg muscle biopsy showing thinned muscle fibers (yellow arrow), characteristic of muscular atrophy in SPS. Note: At the time of biopsy, levels of glutamic acid decarboxylase (GAD)-antibody were elevated. COPD: chronic obstructive pulmonary disease; SPS: stiff-person syndrome

Three months later, he was hospitalized for dyspnea, chest pressure, and diffuse spasms. Clinicians pursued a right lower lobe wedge biopsy to exclude interstitial lung disease, invasive fungal disease, or malignancy in the setting of CVID and COPD; histopathology showed diffuse alveolar architecture destruction consistent with COPD, without interstitial fibrosis or organizing pneumonia. A right lower leg skeletal muscle biopsy was obtained to exclude inflammatory myopathy, given disproportionate weakness and to support the SPS workup; it demonstrated fiber thinning and diffuse atrophy without myositis (Figure 3).

Due to refractory stiffness and recurrent infections, combined IVIG (IVIG dosing: 2 g/kg over three days initially, then 0.7 g/kg every three to four weeks) and rituximab (rituximab dosing: 375 mg/m^2^ weekly × 4, then maintenance every six months) were initiated (December 2022). Within eight weeks of regimen initiation, the patient reported decreased frequency and severity of spasms, improved ambulation tolerance, and fewer respiratory infections. IgG troughs increased from 386 mg/dL to 850 mg/dL, with a reduction in antibiotic courses and no serious infusion reactions throughout.

Approximately 2.5 years after starting rituximab, a motor vehicle accident necessitated spinal fusion, after which exertional dyspnea worsened and supplemental oxygen was required. Over the following two months, IgG troughs became variable, and two uncomplicated pneumonias recurred, which were treated with outpatient antibiotic treatment. To improve pharmacokinetic stability and home-based adherence, therapy was transitioned to subcutaneous immunoglobulin (SCIG) 10% with rHuPH20, with planned monthly monitoring of infections, immunoglobulin levels, and SPS symptom scores. At the two-week follow-up after SCIV, improved IgG stability and symptom control were seen.

## Discussion

The concurrent management of SPS and CVID demands a careful balance-attenuating autoimmune B-cell-mediated neuronal hyperexcitability while preserving residual humoral immunity to prevent serious infections.

**Figure 4:**
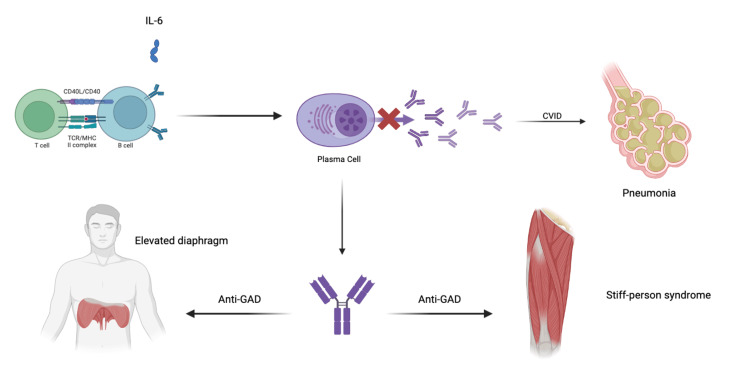
Proposed mechanistic relationship between CVID and SPS. The inability of plasma cells to create appropriate antibodies not only predisposes to infectious diseases but is thought to cause a low level of production of aberrant antibodies, one of which could be an autoantibody to GAD. CVID: common variable immunodeficiency; SPS: stiff-person syndrome; GAD: glutamic acid decarboxylase Image Credit: Samuel Escobar

Although mechanistic data are limited, autoimmunity is common in CVID, and dysregulated B-cell tolerance may predispose to the production of pathogenic autoantibodies, including anti-GAD in SPS [9]. Case-based and cohort reports suggest an enrichment of autoimmune neurologic syndromes among patients with primary antibody deficiencies, but the strength of association and causality remains uncertain [11]. In our patient, CVID-related hypogammaglobulinemia likely contributed to severe fungal pneumonia, while SPS-related respiratory muscle dysfunction (elevated hemidiaphragm) compounded exertional dyspnea. This bidirectional vulnerability underscores the need for integrated neurologic and immunologic management. Although the association between SPS and CVID has not been fully elucidated, current proposed mechanisms describe the aberrant production of antibodies haphazardly and thus increase the risk of autoantibody production [12]; one such target for autoantibody production could be anti-GAD antibodies, as illustrated in Figure 4.

IVIG addresses both conditions via complementary mechanisms. In SPS, IVIG may neutralize pathogenic autoantibodies and modulate Fc-mediated immune pathways, reducing spasms and rigidity [13]. In CVID, immunoglobulin replacement restores protective antibody levels and decreases sinopulmonary infections [14]. Rituximab targets CD20+ B cells, reducing autoantibody production and B-cell antigen presentation, which can ameliorate SPS; however, it risks further depleting humoral immunity [15]. In this case, combining IVIG with rituximab provided dual benefit: clinical improvement in SPS with protection against infections through immunoglobulin replacement. Close monitoring of immunoglobulin levels, infection frequency, and vaccine responsiveness is essential to mitigate over-immunosuppression.

The transition to SCIG with rHuPH20 was a deliberate response to variable IgG troughs and recurrent infections during recovery from spinal surgery. SCIG offers steadier serum IgG exposure, fewer systemic adverse reactions, and home-based administration that may improve adherence [16]. For patients with coexisting SPS and CVID, stable IgG kinetics may help sustain neuromuscular improvement and prevent infectious setbacks, especially when B-cell-depleting therapy is used [17,18].

Although human immunoglobulin with rHuPH20 is not specifically approved for the treatment of SPS or CVID, it has been shown to maintain more consistent IgG levels in those with IgG-consumptive autoimmune conditions, such as chronic inflammatory demyelinating polyneuropathy (CIDP) and idiopathic inflammatory myositis (IIM), and thus offers a potential for improved infection prevention in patients with SPS [19-21]. Moving forward, we would like to trend our patient's serum IgG levels, continue to monitor infection frequency and cause, and evaluate how well this non-traditional treatment regimen decreases the frequency and severity of SPS-exacerbations and CVID-related immunoglobulin deficiency.

Reports describing SPS in the setting of primary immunodeficiencies are scarce, and most focus on single-condition treatment paradigms. Our case reinforces prior observations that IVIG benefits SPS and extends them by showing that, in CVID, rituximab can be deployed safely when coupled with diligent immunoglobulin replacement and surveillance. Prospective data on combined regimens in this dual-diagnosis population are lacking; standardized monitoring of immunoglobulin troughs, infection endpoints, and SPS functional metrics would advance the field.

## Conclusions

In refractory SPS, a targeted immunologic evaluation can reveal coexisting humoral deficiency. When CVID is present, combined IVIG and rituximab may provide functional improvement while maintaining infection control-provided immunoglobulin levels and infectious events are closely monitored. Transitioning from IVIG to SCIG with rHuPH20 can stabilize IgG exposure and sustain clinical gains when trough variability or infections emerge.

This case outlines a therapeutic sequence, IVIG plus rituximab with subsequent SCIG optimization, that addresses both autoimmunity and antibody deficiency, and it highlights respiratory muscle involvement as a contributor to dyspnea in SPS. Multidisciplinary care (neurology, immunology, and pulmonology) and prospective evaluation of combined B-cell-targeted therapy with optimized immunoglobulin replacement are needed to clarify safety, efficacy, and mechanisms linking autoimmunity and hypogammaglobulinemia.

## References

[1] Rakocevic G, Floeter MK (2012). Autoimmune stiff person syndrome and related myelopathies: understanding of electrophysiological and immunological processes. Muscle Nerve.

[2] Ciccotto G, Blaya M, Kelley RE (2013). Stiff person syndrome. Neurol Clin.

[3] Alexopoulos H, Dalakas MC (2013). Immunology of stiff person syndrome and other GAD-associated neurological disorders. Expert Rev Clin Immunol.

[4] El-Abassi R, Soliman MY, Villemarette-Pittman N, England JD (2019). SPS: understanding the complexity. J Neurol Sci.

[5] Tsiortou P, Alexopoulos H, Dalakas MC (2021). GAD antibody-spectrum disorders: progress in clinical phenotypes, immunopathogenesis and therapeutic interventions. Ther Adv Neurol Disord.

[6] Hadavi S, Noyce AJ, Leslie RD, Giovannoni G (2011). Stiff person syndrome. Pract Neurol.

[7] Ortiz JF, Ghani MR, Morillo Cox Á, Tambo W, Bashir F, Wirth M, Moya G (2020). Stiff-person syndrome: a treatment update and new directions. Cureus.

[8] Baker MR, Das M, Isaacs J, Fawcett PR, Bates D (2005). Treatment of stiff person syndrome with rituximab. J Neurol Neurosurg Psychiatry.

[9] Cunningham-Rundles C, Bodian C (1999). Common variable immunodeficiency: clinical and immunological features of 248 patients. Clin Immunol.

[10] Bonilla FA, Barlan I, Chapel H (2016). International Consensus Document (ICON): common variable immunodeficiency disorders. J Allergy Clin Immunol Pract.

[11] Dalakas MC (2023). Therapies in stiff-person syndrome: advances and future prospects based on disease pathophysiology. Neurol Neuroimmunol Neuroinflamm.

[12] Dade M, Berzero G, Izquierdo C (2020). Neurological syndromes associated with anti-GAD antibodies. Int J Mol Sci.

[13] Dalakas MC, Fujii M, Li M, Lutfi B, Kyhos J, McElroy B (2001). High-dose intravenous immune globulin for stiff-person syndrome. N Engl J Med.

[14] Baris S, Ercan H, Cagan HH, Ozen A, Karakoc-Aydiner E, Ozdemir C, Bahceciler NN (2011). Efficacy of intravenous immunoglobulin treatment in children with common variable immunodeficiency. J Investig Allergol Clin Immunol.

[15] Pignolo A, Vinciguerra C, Monastero R (2025). Rituximab in stiff-person syndrome with glutamic acid decarboxylase 65 autoantibody: a systematic review. J Neurol.

[16] Ness S (2019). Differentiating characteristics and evaluating intravenous and subcutaneous immunoglobulin. Am J Manag Care.

[17] Otani IM, Lehman HK, Jongco AM (2022). Practical guidance for the diagnosis and management of secondary hypogammaglobulinemia: a work group report of the AAAAI primary immunodeficiency and altered immune response committees. J Allergy Clin Immunol.

[18] Cinetto F, Neri R, Vianello F (2021). Subcutaneous immunoglobulins replacement therapy in secondary antibody deficiencies: Real life evidence as compared to primary antibody deficiencies. PLoS One.

[19] Bril V, Hadden RD, Brannagan TH 3rd (2023). Hyaluronidase-facilitated subcutaneous immunoglobulin 10% as maintenance therapy for chronic inflammatory demyelinating polyradiculoneuropathy: the ADVANCE-CIDP 1 randomized controlled trial. J Peripher Nerv Syst.

[20] Palermo A, Biancalana E, Bettiol A (2025). Recombinant human hyaluronidase-facilitated subcutaneous immunoglobulin (hf-SCIg) for inflammatory myositis: a multicenter retrospective real-world observational study. Eur J Intern Med.

[21] Jolles S (2013). Hyaluronidase facilitated subcutaneous immunoglobulin in primary immunodeficiency. Immunotargets Ther.

